# The nuclear OXPHOS genes in insecta: a common evolutionary origin, a common cis-regulatory motif, a common destiny for gene duplicates

**DOI:** 10.1186/1471-2148-7-215

**Published:** 2007-11-08

**Authors:** Damiano Porcelli, Paolo Barsanti, Graziano Pesole, Corrado Caggese

**Affiliations:** 1 Dipartimento di Genetica e Microbiologia (DIGEMI), Università di Bari, Italy; 2 Dipartimento di Biochimica e Biologia Molecolare, Università di Bari, Italy

## Abstract

**Background:**

When orthologous sequences from species distributed throughout an optimal range of divergence times are available, comparative genomics is a powerful tool to address problems such as the identification of the forces that shape gene structure during evolution, although the functional constraints involved may vary in different genes and lineages.

**Results:**

We identified and annotated in the MitoComp2 dataset the orthologs of 68 nuclear genes controlling oxidative phosphorylation in 11 Drosophilidae species and in five non-Drosophilidae insects, and compared them with each other and with their counterparts in three vertebrates (*Fugu rubripes*, *Danio rerio *and *Homo sapiens*) and in the cnidarian *Nematostella vectensis*, taking into account conservation of gene structure and regulatory motifs, and preservation of gene paralogs in the genome. Comparative analysis indicates that the ancestral insect OXPHOS genes were intron rich and that extensive intron loss and lineage-specific intron gain occurred during evolution. Comparison with vertebrates and cnidarians also shows that many OXPHOS gene introns predate the cnidarian/Bilateria evolutionary split. The nuclear respiratory gene element (NRG) has played a key role in the evolution of the insect OXPHOS genes; it is constantly conserved in the OXPHOS orthologs of all the insect species examined, while their duplicates either completely lack the element or possess only relics of the motif.

**Conclusion:**

Our observations reinforce the notion that the common ancestor of most animal phyla had intron-rich gene, and suggest that changes in the pattern of expression of the gene facilitate the fixation of duplications in the genome and the development of novel genetic functions.

## Background

As the number of sequenced eukaryotic genomes steadily increases, systematic comparison of closely related species ("phylogenetic shadowing") [1] allows characterization of recent evolutionary events before they are obscured through accumulation of random mutations, while comparison over larger evolutionary distances highlights lineage specific changes. Besides providing a better understanding of many aspects of genome evolution, recent studies based on a comparative genomic-based approach led to significant progress in clarifying the molecular mechanisms that control gene evolution and the origin of the differences in gene structure between eukaryotic species [2], showing that changes in exon-intron structure are largely independent of protein sequence evolution [3]. Comparative analysis is also a powerful tool to identify conserved noncoding sequences essential for regulating gene expression [4-7]. However, investigation of the forces that shape eucaryotic genomes is still significantly hampered by the absence of comprehensive and readily available data on a sufficient number of informative lineages. Studies involving as large as possible a number of different species and gene subsets are needed, because selective pressures may differ significantly not only between different evolutionary lineages but also between particular types of genes [8].

Oxidative phosphorylation (OXPHOS), the primary energy-producing biological process in all aerobic organisms [9], generates ATP using the products of both nuclear and mitochondrial genes (OXPHOS genes); because they encode products organized in the respiratory complexes spanning the inner mitochondrial membrane, OXPHOS genes are subject to specific evolutionary constraints, e.g. because coordinate evolution is required to maintain the stochiometric balance between components of multisubunit complexes [10].

We previously reported [11] the identification of the *D. pseudoobscura *and *A. gambiae *orthologs of a set of *D. melanogaster *genes which are the putative counterparts of human OXPHOS genes [12]. To extend our analysis, we recently identified and annotated the OXPHOS genes orthologs in nine more Drosophilidae genomes, in another Culicidae species (the yellow fever mosquito, *Aedes aegypti*), and in three non Dipteran insect species, i.e. *Bombyx mori *(silkworm), *Apis mellifera *(honeybee) and *Tribolium castaneum *(red fluor beetle), and we have compiled the MitoComp2 dataset [13], that provides an integrated view of the data obtained. Duplicates of the OXPHOS genes, when present in a genome, were also included in the dataset, which at present contains more than 1300 annotated genes. Drawing on this information, we present here a detailed comparative analysis of 68 gene clusters each comprising the putative orthologs of an OXPHOS gene in 11 Drosophilidae species and in five non-Drosophilidae insects.

This study focuses on three aspects of the evolutionary history of the insect OXPHOS genes, i.e. i) conservation of the exon-intron structure during evolution; ii) identification and conservation of a putative regulatory motif specific of genes involved in energy production in insects; iii) origin, fixation in the genome and functional significance of OXPHOS genes duplicates. We also compared the exon-intron structure of insect OXPHOS genes with their counterparts in three vertebrate species (*Fugu rubripes *(pufferfish), *Danio rerio *(zebrafish) and human), and in the cnidarian *Nematostella vectensis *(starlet sea anemone). The last comparison was felt informative because of the pivotal position that cnidarians occupy in metazoan phylogeny [14].

On the whole, our findings further validate the use of interspecific multialignments of orthologous sequences as a powerful tool to identify crucial features that constrain genome evolution. We identified several such features within OXPHOS genes transcriptional units, including known and novel regulatory motifs, splicing sites and previously unidentified genes within genes.

## Results and discussion

### MitoComp2 : a web resource for the comparative analysis of insect OXPHOS genes

The MitoComp2 dataset [13] includes sequence and structural information about the orthologs of 68 *D. melanogaster *OXPHOS genes in a set of sequenced insect genomes diverging enough to make possible the investigation of long term trends in the evolutionary history of these gene. Table 1 lists the 68 OXPHOS genes addressed by our analysis. Beside information on *D. melanogaster*, *D. pseudobscura *and *A. gambiae *OXPHOS genes [11], MitoComp2 annotates the OXPHOS orthologs in 13 additional insects whose genome has been recently sequenced: nine Drosophilidae (*D. simulans*, *D. yakuba*, *D. erecta*, *D. ananassae*, *D. persimilis*, *D. willistoni*, *D. mojavensis*, *D. virilis *and *D. grimshawi*), a second Culicidae (*Aedes aegypti*), and three non-dipteran insects (*Bombyx mori, Apis mellifera *and *Tribolium castaneum*).

**Table 1:** The 68 nuclear OXPHOS genes studied in this work

**Complex**	**Name**	**Subunits**	**Gene product (*D. melanogaster *gene)**
I	NADH ubiquinone oxidoreductase	34	**13 kDa A **(CG8680), **13 kDa B **(CG6463), **15 kDa **(CG11455), **18 kDa **(CG12203), **19 kDa **(CG3683), **20 kDa **(CG9172), **23 kDa **(CG3944), **24 kDa **(CG5703), **30 kDa **(CG12079), **39 kDa **(CG6020), **42 kDa **(CG6343), **49 kDa **(CG1970), **51 kDa **(CG9140), **75 kDa **(CG2286), **B8 **(CG15434), **B12 **(10320), **B14 **(CG7712), **B14.5A **(CG3621), **B14.5B **(CG12400), **B14.7 **(CG9350), **B15 **(CG12859), **B16.6 **(CG3446), **B17 **(CG13240), **B17.2 **(CG3214), **B18 **(CG5548), **B22 **(CG9306), **ACP **(CG9160), **ASHI **(CG3192), **MLRQ **(CG32230), **MNLL **(CG18624), **PDSW **(CG8844), **SGDH **(CG9762), **AGGG **(CG40002), **MWFE **(CG17054).

II	Succinate dehydrogenase	4	**Flavoprotein **(CG17246), **Iron-sulfur **(CG3283), **Cytochrome B560 **(CG6666), **Cytochrome B small subunit **(CG10219).

III	Ubiquinol-cytochrome c oxidoreductase	9	**6.4 kDa **(CG14482), **7.2 kDa **(CG8764), **11 kDa **(Ucrh), **14 kDa **(CG3560), **Iron-sulfur **(CG7361), **Cytochrome C1 **(CG4769), **Core protein 1 **(CG3731), **Core protein 2 **(CG4169), **Ubiquinone-binding protein QP-C **(CG7580).

IV	Cytochrome c oxidase	8	**IV **(CG10664), **Va **(CG14724), **Vb **(CG11015), **VIa **(CG17280), **VIb **(CG14235), **VIc **(CG14028), **VIIa **(CG9603), **VIIc **(CG2249).

V	F0/F1 ATP synthase	13	**Alpha **(CG3612), **Beta **(CG11154), **Gamma **(CG7610), **Delta **(CG2968), **Epsilon **(CG9032), **B **(CG8189), **D **(CG6030), **E **(CG3321), **F **(CG4692), **G **(CG6105), **Coupling factor 6 **(CG4412), **Lipid-binding protein **(CG1746), **OSCP **(CG4307).

**Figure 1:**
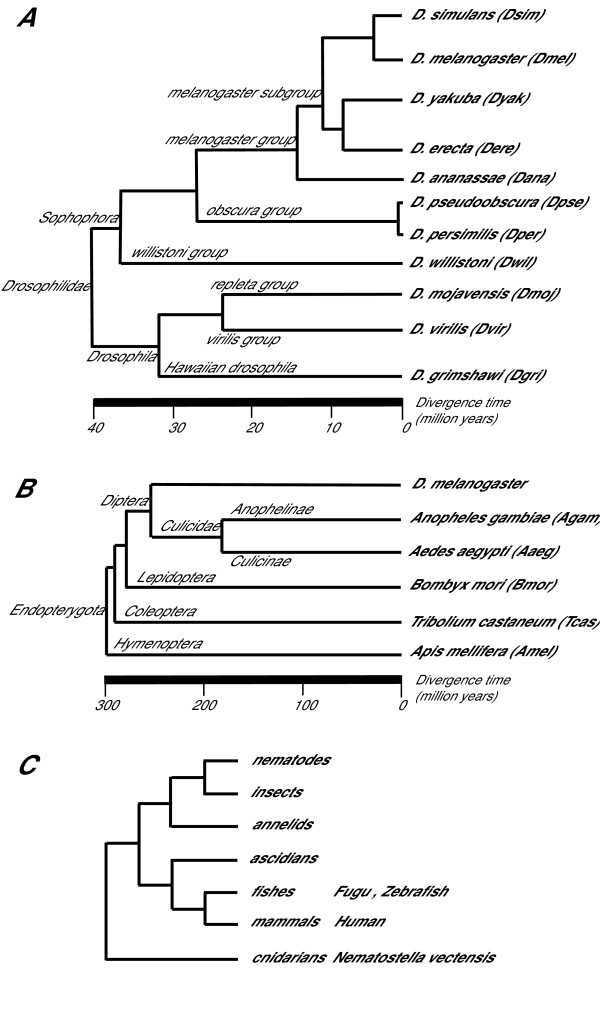
Simplified evolutionary trees showing the phylogenetic relationships of the species addressed in this study. Abbreviations used in the text are in parentheses. (A) Evolutionary relationships of the Drosophilidae species studied. Tree structure and estimated divergence times are from Russo and Takezaki [14]. (B) Simplified insect evolutionary tree. In accord with recent works [17,18] the Hymenoptera are basal to the Coleoptera in the Endopterygota. (C) Global evolutionary tree showing the early divergence of cnidarians and Bilateria.

The Drosophilidae species studied span an evolutionary time of 40–60 Myr [15,16] (Figure 1A), while *A. aegypti *and *A. gambiae *diverged approximately 180 Mya [17]. A simplified evolutionary tree including all insect species studied in this work is shown in Figure 1B. According to most recent phylogenies, Hymenoptera are shown in the figure as basal to Coleoptera in the Endopterygota [18,19].

To identify the putative orthologs of the *D. melanogaster *OXPHOS genes in other insect species we performed whole genome BLAST searches using the CDSs and amino-acid sequences of the *D. melanogaster *genes as queries. Orthology/paralogy relationships were inferred from (1) similarity of gene products, (2) conservation of exon/intron structure, (3) conservation of microsyntenic gene order and (4) evidence from phylogenetic trees. Sequences giving the reciprocal best hits in each genome were considered members of the orthologous gene cluster provided that the BLAST E-value was less than 10^-30 ^and that they could be aligned with the *D. melanogaster *gene over at least 60% of the gene length. According to this criterion, all 68 OXPHOS genes investigated were found to have a counterpart in each insect species studied, except for five genes that were not identified in *A. mellifera *and one in *T. castaneum *possibly because the relevant genomic sequences were incomplete or did not give significant BLAST E-values due to an high level of divergence with the query sequences.

In total, we have annotated 1336 OXPHOS genes of 16 insect species. Table 2 shows a overwiew of the comparison of the OXPHOS genes in the species addressed in this work. Each Mitocomp2 gene record shows the exon-intron structure of the gene, its annotated genomic sequence, the mRNA or CDS sequence and the amino acid sequence of the encoded polypeptide. A textual hyperlink is also provided to a web page containing comparative data on the orthologous genes and their duplications in all Drosophilidae species studied, including comparison of exon-intron structure, alignments of the coding sequences and of the deduced amino acid products, and alignments of the conserved noncoding sequences. Data on the microsyntenic context and phylogenetic relationships of OXPHOS gene duplicates, when present, are also reported.

**Table 2:** Overview of the comparison of the OXPHOS genes

**Species**	Dros *	***Agam***	***Aaeg***	***Bmor***	***Amel***	***Tcas***	***Fugu***	***Zebra fish***	***Human***
	**Orthologous genes**								

No of genes	68	68	68	68	63	67	68	68	68
One exon genes	8	10	7	6	3	9	0	0	0
No of exons^§^	191	183	189	257	235	198	368	369	369
No of introns^§^	122	114	119	187	171	129	300	301	301
No of introns^§ ^per gene	2.0	1.9	1.8	3.0	2.7	2.2	4.4	4.4	4.4
Av. intron size (bp)	173	199	3503	748	204	332	350	1181	2994

	**Gene duplicates**								

Duplication events	34	8	4	7	2	3	25	22	24
One exon genes	121	3	2	3	0	2	0	0	4

*Dros. indicates the 11 Drosophilidae species. ^§ ^Only coding exons and introns between coding exons are considered. Species abbreviations as in Figure 1.

MitoComp2 also contains additional information on the comparison of the non-Drosophilidae OXPHOS genes with their *D. melanogaster *orthologs (taken as representative of the Drosophilidae), providing a link to the multialignment of the coding sequences of the genes compared where the position of the introns is highlighted; a link to the multialignment of the deduced amino acid sequences of the gene products, including their human counterpart; and finally a schematic drawing comparing the exon-intron structure of members of the orthologous gene cluster.

### OXPHOS gene structure evolution in insects

**Figure 2:**
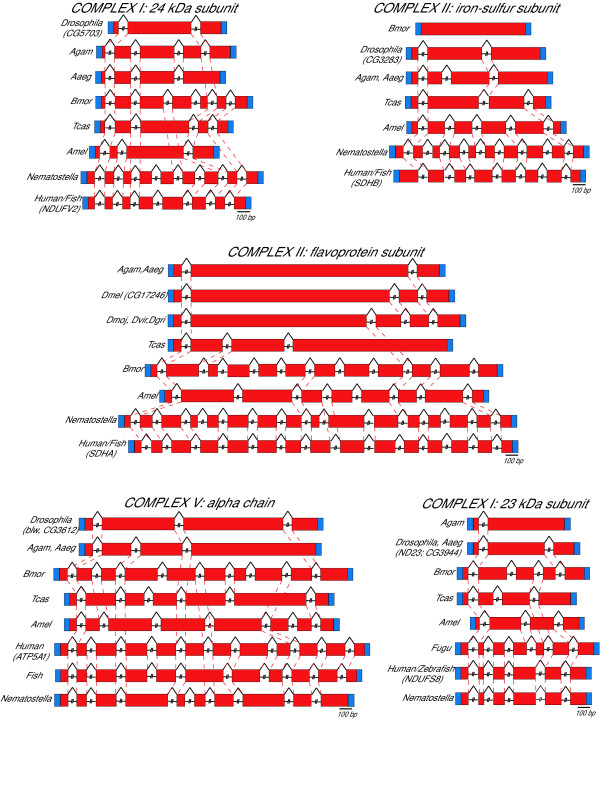
Examples of comparative analysis of the exon/intron structure of orthologous OXPHOS genes. Pre-mRNA are compared. Dashed lines indicate conservation of intron position; UTRs (blue boxes) are not in scale. Species abbreviations as in Figure 1. The sequence alignments on which the figure is based are available at the MitoComp2 web site [13].

Intron sequences are subject to selection not only because they may contain ORFs or form part of coding sequences due to alternative splicing, but also because they can play a regulatory role in transcription or translation, or in maintaining pre-mRNA secondary structure [2]. However, the evolutionary mechanisms and dynamics of intron gain/loss are as yet only incompletely understood. On the assumption that comparison of the structural organization of OXPHOS genes in an informative range of species would prove useful to study the mechanisms shaping gene structure during evolution, we first compared the exon-intron structure of 68 OXPHOS orthologous genes in 11 Drosophilidae species, then we assessed intron gain/loss in the orthologs of these genes in five non Drosophilidae insects. As an example of the results obtained, a visualization of the changes in the exon-intron structure of a few orthologous gene sets during evolution is shown in Figure 2. Similar data for all the gene clusters studied are available at the MitoComp2 website [13].

**Figure 3:**
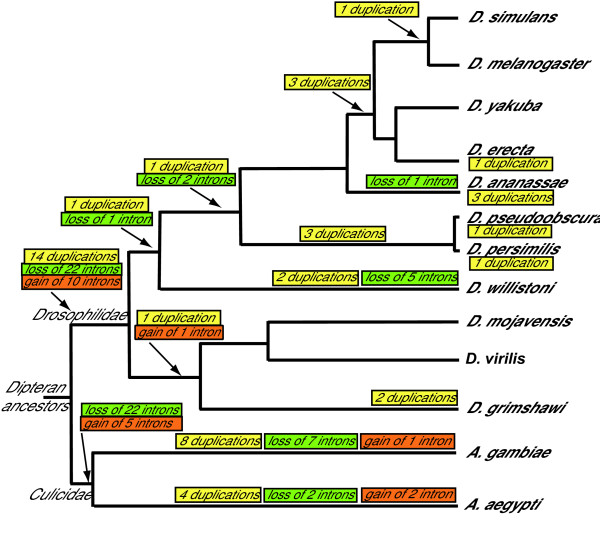
Distribution of the inferred duplication (yellow boxes), intron gain (orange boxes) and intron loss (green boxes) events during evolution of the Drosophilidae and Culicidae species addressed in this work. The branch lengths are not proportional to age.

Table 3 summarizes intron position conservation in the insect species studied. In Drosophilidae, the exon-intron structure of the OXPHOS genes is completely conserved in 61 out of 68 orthologous gene clusters; 117 out of 127 intron positions are conserved at the same nucleotide position throughout the species studied, and only 10 discordant positions in seven different genes were found. Microsyntenic gene order and comparison with non-Drosophilidae insects suggests that nine of the changes are due to intron loss, one to intron gain (Figure 3 and Additional file 1). Eight orthologous gene clusters comprise only intronless genes.

**Table 3:** Pair-wise conservation of intron position in the OXPHOS genes

Species	*Dros*.* (122)	*Agam *(114)	*Aaeg *(119)	*Bmor *(187)	*Amel *(171)	*Tcas *(133)	*Human *(301)
Dros.* (122) vs.	-	70%	73%	77%	63%	61%	59%
*Agam *(114) vs.	75%	-	98%	72%	68%	69%	63%
*Aaeg *(119) vs.	75%	94%	-	81%	66%	68%	59%
*Bmor *(187) vs.	50%	44%	51%	-	54%	53%	46%
*Amel *(171) vs.	45%	46%	46%	59%	-	50%	60%
*Tcas *(133) vs.	56%	69%	61%	74%	65%	-	60%
*Human *(301) vs.	24%	24%	23%	29%	34%	26%	-

*Insecta *(305)	-	-	-	-	-	-	44%

*Dros. indicates the 11 Drosophilidae species. The total number of introns identified in each species is indicated in parentheses. Each number in the table indicates the percentage of the concordant intron positions in the two species identified by the row/column intersection. Species abbreviations as in Figure 1.

Comparison of Drosophilidae and Culicidae indicates descent from a common Dipteran ancestor for members of almost all orthologous gene clusters studied; massive intron loss appears to have occurred indipendently in the two lineages (Figure 3).

Comparison of Dipterans with *B. mori*, *T. castaneum *and *A. mellifera *also indicates descent of non-Dipteran OXPHOS genes from ancestral intron-rich genes that existed before the divergence of insect lineages. 32% of the OXPHOS genes maintain an identical exon-intron structure in all insect genomes studied, while in 19% a discordant intron position was observed in at least one species. In the remaining instances, multiple changes in gene structure were found. In accord with studies suggesting that recombination between genomic sequences and a product of reverse transcription of a processed mRNA is the main mechanism of intron loss in mammals [20], in most cases only a single intron was lost, while the neighboring introns are conserved; in no case a gap was observed in the alignment, and the sequences flanking the lost intron are always strongly conserved.

The amount of both intron loss and intron gain differs strikingly between insect lineages. Using presence of an intron at a given position in a single species only as a criterion to infer intron gain after lineage divergence, we observed one lineage-specific gain event in the 11 Drosophilidae species studied, one in *A. gambiae*, two in *A. aegypti*, 36 in *B. mori*, 27 in *A. mellifera *and 15 in *T. castaneum*. It is of course possible that some of the concordant intron positions are due to independent insertions in different lineages [21,22]; however, this is unlikely to explain all, or even most, observed intron-position correspondences [23].

**Figure 4:**
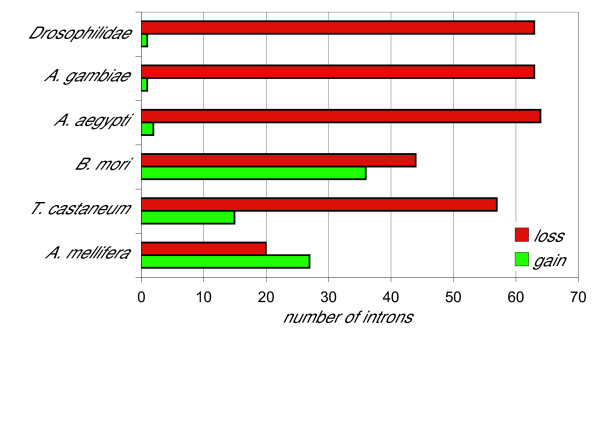
Number of intron gains and losses in the OXPHOS genes of extant insects with respect to the inferred gene structure in a common ancestor.

Assuming that positions conserved in multiple insect lineages and also in Vertebrates (see below) represent retained ancestral introns, the presence of a minimum of 136 introns in the OXPHOS genes of a common ancestor predating the divergence of insect lineages can be inferred; of those introns, 63 were lost in Drosophilidae, 63 in *A. gambiae*, 64 in *A. aegypti*, 44 in *B. mori*, 57 in *T. castaneum *but only 20 in *A. mellifera *(Figure 4 and Table 3).

The number of discordant intron positions observed in *A. gambiae *and *A. aegypti*, compared with the data obtained in Drosophilidae, suggests that the number of intron gain/loss events is not directly related to divergence time. Comparing *A. gambiae *and *A. aegypti*, on 121 total intron positions, 12 discordant positions were observed in 11 genes (Figure 3 and Additional file 2); in nine cases, comparison with other species strongly suggests intron loss; in three, the change in gene structure was most likely due to intron gain.

Overall, our data support a scenario in which extensive intron loss from intron-rich ancestral genes occurred during evolution of most insect OXPHOS genes. Intron gain also appears to have occurred quite often, although much less frequently in Dipterans than in other insect lineages (Figure 4).

### Long term evolution of OXPHOS genes

The tentative scenario inferred from the comparative analysis of OXPHOS gene evolution in insects prompted us to extend our analysis to other, distantly related evolutionary lineages. First, we asked whether insect OXPHOS genes share a subtantial fraction of the intron positions with their orthologs in vertebrates, which would indicate an ancient origin predating the insect-vertebrates evolutionary split. To get information on this question we compared the exon-intron structure of the OXPHOS genes in Fugu, zebrafish and humans with each other and with their insect orthologs; the human/fish comparison is highly informative because among sequenced vertebrate genomes fish genomes are the most distantly related available for comparison with humans (the last common ancestor of fish and humans dates back 400–450 Myr, a divergence time substantially longer than the time of divergence of the insect species studied in this work).

Conservation of intron position, exon phase and exon length between insect OXPHOS genes and their counterparts in Fugu, Zebrafish and human strongly suggest descent from a common intron-rich ancestor in 55 out of 68 orthologous gene clusters.

In accord with recent work showing that the exon-intron structure of the gene is highly conserved throughout Vertebrates [24], very few changes in the organization of the OXPHOS genes were observed between Fugu and zebrafish, and, strikingly, between fish and humans. We found only two gene structure changes in vertebrates: the sequence of a coding exon of the NDUFS8 gene, encoding the 23 kDa subunit of Complex I, is interrupted in Fugu, but not in human or zebrafish, while an exon of the ATP5A1 gene, encoding the alpha chain of Complex V, is interrupted in human, but not in fish (Figure 2).

The last column of Table 3 reports the number of intron positions shared by human OXPHOS genes with their orthologs in the insect species studied. In accord with recent findings suggesting that the honeybee genome is the slowest evolving of the insect genomes so far sequenced [25], among the insect species examined *A. mellifera *shares the largest number of OXPHOS gene intron positions with Vertebrates: out of 171 total introns identified in the OXPHOS genes of *A. mellifera*, 60% are conserved at the same position in their human counterparts, while 34% of 301 intron position in human OXPHOS genes are shared with *A. mellifera*.

Finally, to extend the evolutionary history of the OXPHOS genes beyond the recent report that a significant fraction of human introns are shared with the genome of annelids [26] and therefore must predate the bilaterian radiation, we thought of interest to compare the exon-intron structure of 15 human OXPHOS genes with their orthologs in the cnidarian *N. vectensis *(as shown in Figure 1C, separation of the cnidarian lineage from Bilateria predates the Urbilaterian ancestor). This comparison revealed that *N. vectensis *shares 80% of the intron OXPHOS gene positions with Vertebrates, while 84% of the human intron positions are shared by *Nematostella *(Figure 2 and MitoComp2 web site [13]). Thus, these introns appear to have been present in very ancient ancestral genes predating even the cnidarian/Bilateria divergence, and the remarkable genetic complexity of Nematostella [14], toghether with the high level of conservation of intron positions between Cnidarians and Vertebrata, makes questionable a correlation between morphological complexity and gene structure.

### Specific constraints on conservation of individual introns

Specific constraints may act on individual introns, favoring their conservation during evolution. Unsurprisingly, and in agreement with our previous report concerning a more limited OXPHOS genes sample [11], the exon-intron structure and the alternative splice forms of the orthologous genes encoding the NADH-ubiquinone oxidoreductase acyl carrier protein (*D. melanogaster mtacp1*, *CG9160*) [27]) and the ATP synthase epsilon chain (*sun*, *CG9032*) [12]), are strictly conserved in all insects studied, as shown by genomic structure comparison, alignment of splice variants and EST mapping (see the gene records in the Mitocomp2 dataset [13]). As suggested by Fedorova and Fedorov [28], conservation of the first intron at the 5'end of many genes also appears to be under stringent functional constraints. Because the first exon of most OXPHOS genes encodes a mitochondrial import signal and is usually much less conserved than other coding exons [29,30], its alignment with orthologous regions is often problematic, and conservation of the first 5' intron position can only be inferred from phase conservation and exon length. With this caveat, the conservation at this position is striking: no loss of this intron was observed in 56 out of 68 orthologous gene clusters, although our analysis necessarily underestimates conservation at this position, since it can only address introns that interrupt the CDS because conservation of introns in the 5' UTR, that usually is hardly alignable with orthologous sequences, cannot be unambigously shown.

As discussed in the next section, conservation of the first 5' intron in OXPHOS genes may have important functional implications depending on the position of the nuclear regulatory gene motif (NRG) [31]: only 13 intronless genes were found out of a total of 283 genes belonging to 48 orthologous gene clusters in which the energy regulatory motif is usually located in the first intron of the coding sequence; on the other hand, when the NRG motif is located upstream of the CDS (as it is in 18 OXPHOS gene clusters), the frequency of one-coding-exon genes is almost 25% (27 out of 107).

### Conservation of regulatory elements

The regulation of eukaryotic gene expression is a process involving many different control mechanisms, including chromatin structure and cis-regulatory DNA sequences that bind specific proteins [32]; recent observations emphasize the importance of intergenic and intronic sequences in regulating transcription [33]. Cross-species DNA sequence comparison is an excellent tool for identifying these biologically important elements, because the level of evolutionary conservation is correlated to the extent of functional constraints.

We used multialignment footprinting [34] and DNA pattern discovery programs [35,36]) to identify conserved motifs in noncoding sequences of the OXPHOS genes in 11 Drosophilidae species, two Culicidae (*A. gambiae *and *A. aegypti*), and three non-dipteran insects (*B. mori*, *A. mellifera *and *T. castaneum*).

We focused our attention primarily on the genomic regions that in *D. melanogaster *contain the nuclear respiratory gene element (NRG), a palindromic 10-bp motif (RTTAYRTAAY) shared by all nuclear OXPHOS genes listed in Table 1 and by many other nuclear genes involved in the biogenesis and function of the mitochondrion [31]. In *D. melanogaster*, most NRG elements are located 160–280 bp downstream of the transcription start site, most often within an intron.

**Figure 5:**
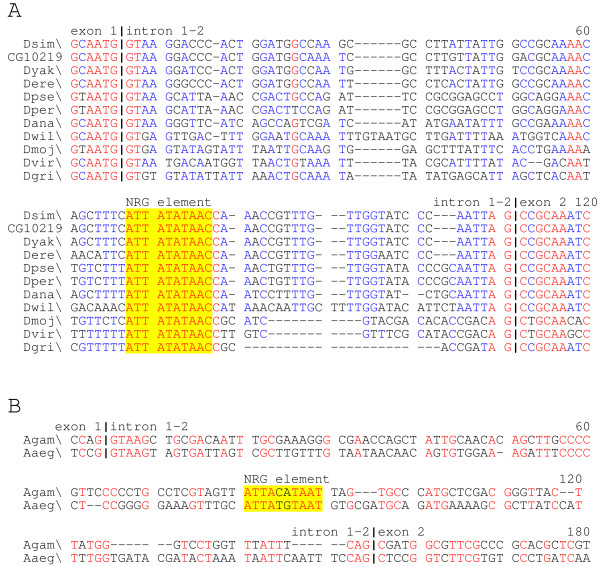
Multiple alignment showing sequence and positional conservation of the NRG element in intron 1–2 of the succinate dehydrogenase [ubiquinone] cytochrome B small subunit gene in Drosophilidae (A) and in *A. gambiae *and *A. aegypti *(B). Alignments are based on Multalin alignments [34] using default parameters for DNA sequences. Red indicates conservation throughout the species examined; blue indicates lesser but still significant conservation. NRG motifs are highlighted in yellow; vertical bars indicate exon/intron boundaries.

The criterion for scoring the conserved NRG elements was presence of a match to the 8 bp core sequence, allowing for substitutions that maintained the consensus structure of the element, which we assume necessary for its function (see also [31]). We searched stretches of about 1000 bp, both upstream and downstream of the TSS, for the presence of the element. With the exception of the orthologs of the *D. melanogaster CG7610 *ATPsyn-gamma gene, containing a conserved variant derivative of the NRG motif in all the species examined, NRG elements were identified in all OXPHOS genes annotated. Interestingly, significant conservation of additional nucleotides flanking the standard 10 bp NRG consensus appears to be a specific feature of different OXPHOS gene clusters. Such extension of the NRG motif beyond the consensus "core" may play a role in the modulation of the expression of individual OXPHOS genes. MitoComp2 [13] shows the relevant features of the NRG elements identified in Drosophilidae, i.e. their consensus sequence, position in the transcriptional unit, and distance from the ATG start site. Links are provided to the alignments of the genomic regions encompassing the elements. As a typical example of the results obtained, Figure 5A shows the multialignment of the NRG-containing intronic sequences of the Drosophilidae orthologs of the *D. melanogaster CG10219 *gene encoding the cytochrome b small subunit of complex II.

Multiple NRG motifs are present in several genes: in 13 orthologous genes clusters, genes containing at least two NRG were found. The number of copies per gene of the element is variable, but is generally conserved between orthologs. An extreme case is the *D. melanogaster CG1746 *gene, encoding the lipid-binding protein P1 of ATP synthase: in the introns of this gene, and in its orthologs in other Drosophilidae species, seven copies of the element were identified.

The NRG element was always found downstream of the transcription start site, in 53 out of 68 orthologous gene clusters within the first intron of the gene. In all other cases, with a single exception, it is located either in the second intron or in the putative 5' UTR sequence. Only in the *CG6666 *gene cluster, encoding the cytochrome b560 subunit of complex II, the NRG element is located in a coding exon.

To study the conservation of the NRG element over long evolutionary times, we also searched noncoding regions of homologous OXPHOS genes of *A. gambiae *and *A. aegypti*, and of *B. mori*, *A. mellifera *and *T. castaneum*. Notwithstanding a divergence time between *A. gambiae *and *A. aegypti *of approximately 180 Mya, pairwise alignment and comparison with orthologous noncoding Drosophilidae sequences identified conserved NRG elements in most OXPHOS genes of these species, often with conservation of the subgenic localization (see Figure 5B for an example, and the MitoComp2 web site [13]), and it was possible to define a Culicidae NRG consensus for almost all OXPHOS genes.

**Figure 6:**
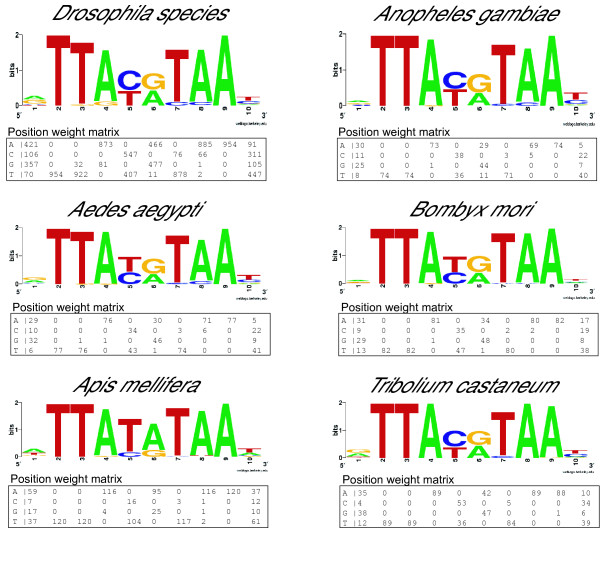
Sequence logo and position weight matrix (PWM) of the OXPHOS gene NRG motif in insects. Logos and PWMs were created, with the Weblogo [57] and Consensus [36] softwares respectively, from multialignments of all the NRG elements identified in each species.

NRG sequences were also found in the OXPHOS genes of the three non-dipteran insect species studied. In *A. mellifera*, the average number of NRG elements per gene is higher than in other insects (in 41 out the 63 *A. mellifera *OXPHOS genes analyzed more than a single NRG element is present), and a TTATATAA NRG sequence is often observed, presumably because the *A. mellifera *genome is more (A+T)-rich than other insect genomes [25]. The list of the all NRG elements found in insect species examined, their position within the transcriptional unit and their distance from the beginning of the CDS (ATG) are shown in MitoComp2 [13]. Figure 6 displays a logo representation of the NRG consensus in these species and the associated position weight matrix.

**Figure 7:**
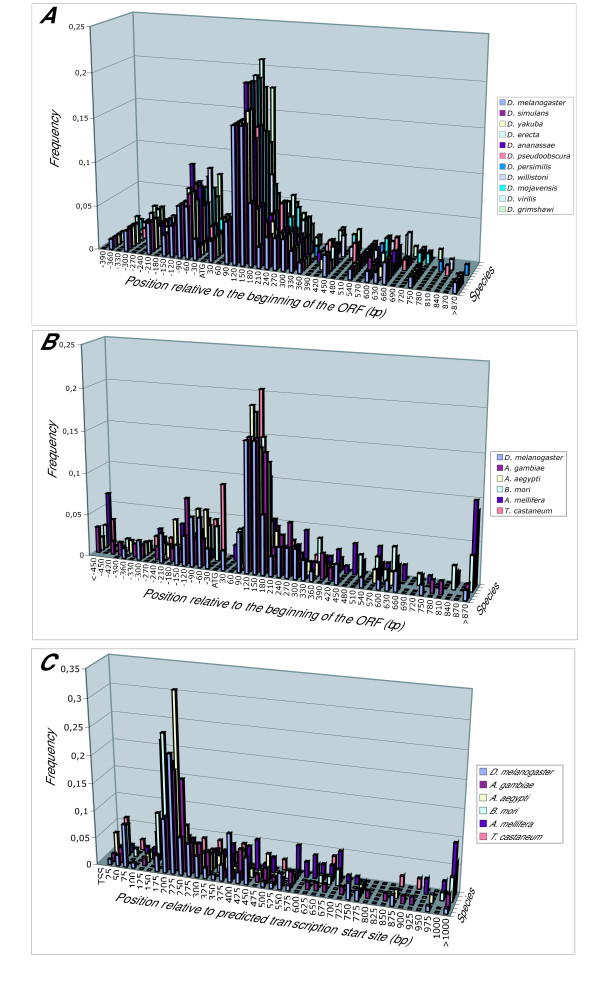
Localization of the NRG motifs in the OXPHOS genes of Drosophilidae and other insect species studied. (A) Distribution relative to the beginning of the ORF in Drosophilidae; (B) distribution relative to the beginning of the ORF in non-Drosophilidae insects; (C) distribution relative to the predicted transcription start site.

The distance between NRG elements and the beginning of the CDS (ATG) is strictly conserved among orthologous Drosophilidae genes (Figure 7A) and is also generally conserved in the non-Drosophilidae insect species studied (Figure 7B). When the putative transcription start site of the gene could be inferred from EST sequences present in the public databases, a strong bias for a location of the NRG element approximately 200 bp downstream of the transcription start site was also observed (Figure 7C). In our opinion, this suggests a critical spatial relationship between the NRG position in the transcriptional unit and the nucleosomal structure of the chromatin, as could be expected if chromatin-domain related mechanisms were implied in controlling the expression of OXPHOS genes.

Although NRG elements represent the most conserved noncoding sequences in orthologous OXPHOS genes, multispecies alignments also identified other significantly conserved elements with a tendency to maintain the same subgenic position, at least in Drosophilidae. Some of these sequences are strictly conserved in several OXPHOS gene clusters; others are specific of a single gene cluster (see Mitocomp2 dataset [13]). As these sequences are almost certainly subject to strong functional constraints, our findings further validate the use of intraspecific phylogenetic comparison to identify novel candidate regulatory elements, although there is increasing evidence of sequences with a cis-regulatory function that exhibit little if any primary sequence conservation, and cannot therefore be identified by multispecies DNA aligment [37].

We did not attempt a systematic study of the functional significance of the non-NRG conserved sequences in the non coding regions of insect OXPHOS genes. However, we would like to report an intriguing example of the interesting insights into the mechanisms shaping genome evolution that data mining the information available in the Mitocomp2 will hopefully provide in the future.

The *CG1746 *gene, encoding the lipid-binding protein P1 of ATP synthase, contains, besides seven highly conserved copies of the NRG element, at least 15 conserved DNA blocks of various lengths scattered throughout its non coding sequences. Eight such DNA stretches, strictly conserved in all the Drosophilidae species studied, are located within the intron at the 3' end of the gene (see the *CG1746 *gene entry in Mitocomp2 dataset [13]). A FlyBase [38] search revealed that in *D. melanogaster *this intron encompasses the untranslated Ribonuclease P RNA gene (RNaseP:RNA), and the sequences highly conserved in this intron are known to be crucial for the ribozyme function [39]. Intriguingly, a search for homologous sequences in *A, gambiae*, *A, aegypti*, *A. mellifera*, *B. mori *and *T. castaneum *indicated that in these insects the orthologous intron does not contain the Ribonuclease P RNA gene, which is instead found within an intron of other, unrelated genes, different in each species (data not shown).

### OXPHOS gene duplications

After previously identifying several duplicates of OXPHOS genes in *D. melanogaster*, *D. pseudoobscura *and *A. gambiae *[11], we have now asked the question whether annotation of OXPHOS genes duplicates in a greater number of insect species at various levels of divergence could provide further information on the forces that have shaped the evolutionary history of this functionally essential set of genes. We found one or more paralogs of 22 different OXPHOS genes in the Drosophilidae species studied. All the identified duplicates of OXPHOS gene appear to be true functional genes, since all present intact ORFs. Assuming that duplicates found in different microsyntenic contexts originated from independent duplication events, 34 independent duplication events would be sufficient to explain all the observed duplicates.

Not surprisingly, since retroposition is probably the most important mechanism of gene duplication and eventual evolution of novel genetic functions [40], of the 34 duplication events inferred 26 almost certainly were retropositional events originating duplicates that are intronless or possess only a very few introns, presumably subsequently acquired. In contrast, four of the events originated duplicates that maintain the intron/exon structure of the parental gene, and so were most probably segmental duplication events. There is no sufficient evidence to assume either mechanism for the remaining four duplication events.

Interestingly, seven of the independent events originated duplicates within introns of other genes, in support of the suggestion that retrocopies can become functional genes by exploiting the regulatory elements and the open chromatin state of neighboring transcriptional units [41]. Presence of the duplication in all the species studied, conservation of microsyntenic gene order and evidence from phylogenetic trees suggest that 14 of the duplication events occurred before, and 20 after the Drosophilidae speciation (Figure 3).

We also found duplications of the OXPHOS genes in non-Drosophilidae. Eight independent duplication events were observed in *A. gambiae*, four in *A. aegypti *(assuming for convenience that in this species the 80 or more copies of the *Aaeg/CG4692 *gene, encoding the f chain of ATP synthase, originated from a single amplification event), seven in *B. mori*, two in *A. mellifera *and three in *T. castaneum*. Seven of these duplications involve genes also duplicated in Drosophilidae, so, although pair-wise orthology can not be reliably assigned between duplicates in Drosophila and in other insects, they were probably present in a common ancestor of all the insect species adressed in this work.

It should be noted that the number of OXPHOS gene duplication events inferred to have occurred in Drosophilidae is significantly higher than in any of the other insect lineages examined. This fact could indicate the existence in Drosophilidae of special mechanisms favoring the fixation of OXPHOS gene paralogs in the genome, or, together with the high number of intron gain/loss events in Drosophilidae reported in a previous section, indicate an especially high level of retrotranscriptional activity in this lineage.

In *D. melanogaster *and in *A. gambiae*, OXPHOS gene duplicates are expressed at a much lower level than their parent genes, as inferred by the abundance in the public databases of ESTs derived from their transcripts; moreover, in *D. melanogaster *they exhibit a strongly testis-biased pattern of expression [11]. Based on this data, we suggested that acquiring a new pattern of expression could be required to maintain a duplicate copy of certain genes in the genome. In support of this hypothesis, in an EST library from *D. yakuba *testes (WashU Drosophila Yakuba EST Project) the expression of OXPHOS gene duplicates is also strongly testis-biased (not shown). That similar mechanisms could favor fixation of gene duplications not only in insects, but also in other organisms is suggested by the independently formulated "out of the testes" hypothesis [41], proposing that in primates functional retrogenes are initially expressed in testes and only later evolve different expression patterns and potentially novel genetic functions.

The study of the conservation of the NRG putative regulatory element in insect OXPHOS genes presented in this paper (see above) provides intriguing evidence suggesting a possible mechanism to maintain OXPHOS gene duplications in the genome. In total, we have identified 215 OXPHOS gene duplicates in the insect species studied. In 214 out of the 215 cases we found one or more NRG elements only in one of the identified paralogs. Conservation of microsynteny (where possible to ascertain, i.e., in Drosophilidae) and of the structural organization of the gene strongly suggest that the genes maintaining the NRG element are the direct phylogenetic derivatives (true functional orthologs) of the ancestral insect genes responsible for the basic housekeeping function of energy production. On the other hand, the NRG motif is absent (or, as shown in the examples of Additional file 3, sharply diverges from the consensus) in almost all OXPHOS gene duplicates that have achieved long-term fixation in the genome. While the absence of the NRG motif is of course expected in duplicates originated by retroposition of genes in which the element is located in an intron, it suggests preferential loss due to selective constraints in duplicates originated by segmental duplication, or by retroposition from the 26 genes in which it is in the 5'UTR. The single exception showing conservation of a standard NRG element in the 5'UTR of two paralogs of the same gene concerns a duplicate of the *mtacp1 *gene, encoding the acyl carrier protein of complex I, which is found in *D. persimilis *but not in its sister species *D. pseudoobscura*. The 100% identity shared not only in the CDS but also in the UTRs regions with one of the mRNAs generated by the alternative splicing of the *mtacp1 *gene transcript, the lack of the introns and of the promoter region and a target-site duplication of eight bp flanking the duplicate suggest that it derives from one of the more recent retroposition events documented to date (see the *mtacp1 *gene entry in MitoComp2 dataset [13]).

Conservation of the NRG element, and probably of its localization in the transcriptional unit, are evolutionary constraints expected to act in a specific manner on OXPHOS genes; however, we would like to suggest that loss of regulatory elements and consequent changes in the pattern of expression of the gene could be a general mechanisms that facilitates the fixation of duplications in the genome when, as in the case of genes encoding products that are part of multiprotein complexes, the presence of multiple gene copies with the original pattern of expression would be deleterious [42]. In turn, this could allow the development of novel genetic functions that is usually assumed to be the main evolutionary advantage of gene duplication [43].

## Conclusion

We have cataloged the orthologs, as identified by sequence homology, conservation of microsynteny and structural organization, of 68 nuclear genes that control oxidative phoshorylation in 11 Drosophilidae species whose genomic sequencing has been recently completed, and in five non-Drosophilidae insect species, and compiled a web-based dataset, MitoComp2 [13], containing all data on which this paper is based and available online. Our results indicate that a common ancestor of the insect lineages examined possessed intron rich OXPHOS genes and that extensive intron loss occurred during evolution; lineage-specific intron gain also occurred, least frequently in Dipterans. Furthermore, comparison with the very distantly related vertebrate and cnidarian lineages shows that many of the OXPHOS genes introns already existed in ancestral genes predating the cnidarian/Bilateria evolutionary split.

Comparative analysis also suggests that conservation of the nuclear respiratory gene element, constantly conserved in the OXPHOS gene orthologs of all the insect species examined, has played a key role in the evolution of the insect OXPHOS genes. Furthermore, we found one or more paralogs of 22 different OXPHOS genes in the Drosophilidae species studied, and showed that only the functional orthologs of the ancestral insect genes responsible for the basic housekeeping function of energy production maintain the NRG element, while their paralogs, either originated by retrotranscription or by segmental duplication either completely lack the NRG element or possess only presumably non-functional relics of the motif. Based on this data, we suggest that changes in the pattern of expression of the gene (as testis-specific expression) could facilitate the fixation of duplications in the genome and in turn the development of novel genetic functions.

## Methods

BlastN and TBlastN [44] searches of contigs, scaffolds and ESTs from FlyBase [38] were performed using *D. melanogaster *OXPHOS CDSs and peptides listed in the MitoDrome database [12] as queries to identify orthologous OXPHOS genes and their duplications in Drosophilidae and other insect genomes.

Sequence sources: *D. erecta*, *D. ananassae*, *D mojavensis*, *D. virilis *and *D. grimshawi *were sequenced by Agencourt, *D. simulans *and *D. yakuba *were sequenced at Washington University, *D. persimilis *and *A. aegypti *were sequenced by the Broad Institute, *D. willistoni *was sequenced by TIGR, *D. melanogaster *was sequenced by the Berkeley Drosophila Genome Project and Celera [45], *D. pseudoobscura *[46], *T. castaneum *and *A. mellifera *[25] were sequenced at Baylor, *A. gambiae *was sequenced by the Anopheles Genome Consortium [47], *B. mori *was sequenced at the Southwest Agricultural University [48] and by the Silkworm Genome Research Program [49].

Contigs, scaffolds and ESTs from the National Center for Biotechnology Information (NCBI) [50] and StellaBase [51] were searched using the human OXPHOS peptides from Swiss-Prot [52] to identify orthologous OXPHOS genes and their duplications in *F. rubripes*, zebrafish (*D. rerio*) and *N. vectensis*. Additionally, single-trace sequences were screened at the TraceSite of NCBI [50] using MEGABlast and were assembled manually. Human genomic and mRNA sequences were retrieved from Ensembl [53].

Duplicate gene pairs within a genome were identified as best reciprocal hits with an E-value of less than 10^-20 ^in both directions in a TBLASTN search using the default parameters. For convenience, each newly identified insect gene is indicated in this paper by a term comprising the abbreviation of the species followed by the CG number of its counterpart in *D. melanogaster*.

Multialignments of amino acid, and of coding and noncoding sequences and visualization of dendrograms were obtained using the MultAlin 5.4.1 software [34] from the MultAlin server [54].

The genomic sequence of each gene identified was searched manually for exon-intron boundaries and the predicted transcribed sequence was reconstructed *in silico*. All insect genomic, mRNA/CDS and amino acid sequences utilized for this study are archived at the MitoComp2 web site [13]; the vertebrate and *Nematostella *genomic sequences recovered are available on request from C.C.

To identify NRG motifs and other conserved elements in noncoding sequences of Drosophilidae OXPHOS genes, we aligned members of each orthologous gene cluster, manually defined exon-intron boundaries, and searched for DNA stretches maintaining high consensus in all the Drosophilidae species studied. Pair wise alignment and comparison with orthologous Drosophilidae sequences was used to identify conserved NRG elements in noncoding sequences of *A. gambiae *and *A. aegypti *OXPHOS genes.

To identify NRG motifs in *B. mori*, *A. mellifera *and *T. castaneum *OXPHOS genes we used the Weeder pattern discovery program [35] and the Regulatory Sequence Analysis Tools from the RSAT server [55]. Position weight matrices (PWM) were created with the Consensus software from the RSAT server. The graphical representations of NRG motifs as sequence logos [56] were generated using WebLogo [57].

## Authors' contributions

DP and CC carried out initial study design, conducted the data mining and contributed to writing the manuscript. PB and GP supervised and contributed to writing the manuscript. All authors read and approved the final manuscript.

## Supplementary Material

Additional file 1 Intron gain/loss in the Drosophilidae OXPHOS genes. Intron gain/loss in the Drosophilidae OXPHOS genes determined by comparing the pre-mRNA structures. Click here for file

Additional file 2 Intron gain/loss in the Culicidae OXPHOS genes. Intron gain/loss in the Culicidae OXPHOS genes determined by comparing the pre-mRNA structures. Click here for file

Additional file 3 Relics of NRG elements in duplicated OXPHOS genes. The regions of the putative parental gene encompassing the standard NRG motif(s), highlighted in yellow, are aligned with the orthologous regions of the duplicate. Click here for file
