# Supplementary material for: The nuclear OXPHOS genes in insecta: a common evolutionary origin, a common cis-regulatory motif, a common destiny for gene duplicates
Source: BMC Evol Biol. 2007 Nov 8;7:215. doi: 10.1186/1471-2148-7-215 (PMC2241641; doi:10.1186/1471-2148-7-215)
Supplement: Additional file 3 — Relics of NRG elements in duplicated OXPHOS genes. The regions of the putative parental gene encompassing the standard NRG motif(s), highlighted in yellow, are aligned with the orthologous regions of the duplicate. [file 1471-2148-7-215-S3.doc]

NADH-ubiquinone oxidoreductase 18 kDa subunit

1 5’UTR 60

Dpse\CG12203 ACAAAACCGATTGATTTCTGTTCCAAATTTTACGTA------ATTCTACCTGCGA---AA

Dper\CG12203 ACAAAACCGATTGATTTCTGTTCCAAATTTTACGTA------ATTCTACCTGCGA---AA

Dpse\dupl CAAGAGATATTTGTGTTCCGTTCCAAATTGTACGTACGTAAGATTGAAATTGCGAGAGAA

Dper\dupl CAAGAGATATTTGTGTTCCGTTCCAAATTGTACGTACGTAAGATTGAACTTGCGAGAGAA

61|->CDS exon**|**intron 120

Dpse\CG12203 **ATG**TCGCTTATACGCCAAGTAATGTGCAGGACTACA------TCCTTTCAGCT**|**GTAAGTG

Dper\CG12203 **ATG**TCGCTTATACGCCAAGTAATGTGCAGGACTACA------TCCTTTCAGCT**|**GTAAGTG

Dpse\dupl **ATG**TCGCTCCTACGTCGAGTAATGTGCAGGACTACCGCCACCTCCTTGCAGTT**|**GTAGGTG

Dper\dupl **ATG**TCGCTCCTACGTCGAGTAATGTGCAGGACTACCGCCACCTCCTTGCAGTT**|**GTAGGTG

NADH-ubiquinone oxidoreductase 49 kDa subunit

1|->CDS 60

Agam\CG1970 **ATG**GCATTCAGCGTGCTGAACACGGTGGCGAAGCGCACCGCCGCGAACGTTTATGTAACC

Agam\dupl **ATG**GCATTCTGTGTGTTAAACGCCGCAGCAAAGCGAACACCAACGACTGTTTACGTAGTC

61 120

Agam\CG1970 GGCGGCGGGCTGCTGAAAAATGTCGCCGCTTTATGTAACGGGCA---GCAGCCGGCCCGG

Agam\dupl GGTCGTGGGCTGCTGCAAAATGTCACCGCTCCGTACCGATTGCATCAGCAGCCAGCCCGT

ATP synthase epsilon chain

1|->CDS exon**|**intron  60

Agam\CG9032 **ATG**GCCGCATGGAGAGCTGCTGGATTGAA**|**GTGAGTTTGTGGCACAGGCAGAGGGACGGCG

Agam\dupl **ATG**GCTGCCTGGAGAACTGCCGGACTGAA**|**GTGAGTTCGAGG-------------------

61 120

Agam\CG9032 GCTGCCCCTCTTCAGTGTGTGTGCGCTCGGGCAACCATTGTTATGTAACCGAGTTGTGCT

Agam\dupl ------------------GTGATCGATGGTGCAA--------ATGGTGCCAATTTAT-CT

121 intron**|**exon 180

Agam\CG9032 AATTGTTTTGCCGTTTTTTTGTTCGCCTTTTTAG**|**CTACATTAACTACTCCAACATTGCCG

Agam\dupl AAATGTTTTGCCGCATGATTGTTCGCCTTTTTAG**|**CTACATCAACTATTCCAACATTGCCG

NADH-ubiquinone oxidoreductase 15 kDa subunit

1 60

Bmor\CG11455 ACCAGAACCTAAAATGTTTACGTAATTATTAACCTAAGTGTATGAAAATCAAAAAAGGTT

Bmor\dupl GTATAATCTATGAATATAATCGTGATTTACATATTT--TTTTCGAATTTAAAATAAAATA

61 5’UTR|->CDS 120

Bmor\CG11455 TGTATGCATTTATTTCAGAAAACATC**ATG**---TCTTTGTCGCCGTTCTTTCGCTCACCGT

Bmor\dupl TCTATAATTTTAATTAATTGAT-ATC**ATG**GACACTATTTCTCCATTCTTCCGTTCACCGT
